# Characterization of Primary Cilia in Osteoblasts Isolated From Patients With ADPKD and CKD

**DOI:** 10.1002/jbm4.10464

**Published:** 2021-02-23

**Authors:** Renata C Pereira, Berenice Y Gitomer, Michel Chonchol, Peter C Harris, Kathleen J Noche, Isidro B Salusky, Lauren V Albrecht

**Affiliations:** ^1^ Department of Pediatrics David Geffen School of Medicine at UCL Los Angeles CA USA; ^2^ Department of Medicine, Division of Renal Diseases and Hypertension University of Colorado Anschutz Medical Campus Aurora CO USA; ^3^ Division of Nephrology and Hypertension Mayo Clinic Rochester MN USA; ^4^ Department of Biological Chemistry David Geffen School of Medicine at UCLA Los Angeles CA USA

**Keywords:** AUTOSOMAL DOMINANT POLYCYSTIC KIDNEY DISEASE, BONE, CHRONIC KIDNEY DISEASE, OSTEOBLASTS, POLYCYSTIC KIDNEY DISEASE, PRIMARY CILIA

## Abstract

Autosomal dominant polycystic kidney disease (ADPKD) is the most common inherited cause of chronic kidney disease (CKD) and leads to a specific type of bone disease. The primary cilium is a major cellular organelle implicated in the pathophysiology of ADPKD caused by mutations in polycystin‐1 (*PKD1*) and polycystin‐2 (*PKD2*). In this study, for the first time, cilia were characterized in primary preosteoblasts isolated from patients with ADPKD. All patients with ADPKD had low bone turnover and primary osteoblasts were also obtained from patients with non‐ADPKD CKD with low bone turnover. Image‐based immunofluorescence assays analyzed cilia using standard markers, pericentrin, and acetylated‐α‐tubulin, where cilia induction and elongation were chosen as relevant endpoints for these initial investigations. Osteoblastic activity was examined by measuring alkaline phosphatase levels and mineralized matrix deposition rates. It was found that primary cilia can be visualized in patient‐derived osteoblasts and respond to elongation treatments. Compared with control cells, ADPKD osteoblasts displayed abnormal cilia elongation that was significantly more responsive in cells with *PKD2* nontruncating mutations and *PKD1* mutations. In contrast, non‐ADPKD CKD osteoblasts were unresponsive and had shorter cilia. Finally, ADPKD osteoblasts showed increased rates of mineralized matrix deposition compared with non‐ADPKD CKD. This work represents the first study of cilia in primary human‐derived osteoblasts from patients with CKD and patients with ADPKD who have normal kidney function, offering new insights as bone disease phenotypes are not well recapitulated in animal models. These data support a model whereby altered cilia occurs in *PKD‐*mutated osteoblasts, and that ADPKD‐related defects in bone cell activity and mineralization are distinct from adynamic bone disease from patients with non‐ADPKD CKD. © 2021 The Authors. *JBMR Plus* published by Wiley Periodicals LLC. on behalf of American Society for Bone and Mineral Research.

## Introduction

Autosomal dominant polycystic kidney disease (ADPKD) represents the most common monogenic cause of end‐stage kidney disease (ESKD)^(^ ^1^, ^2^ ^)^ and is driven predominately by mutations in polycystic kidney disease genes, *PKD1* and *PKD2*, which encode primary cilium proteins polycystin‐1 (PC1) and polycystin‐2 (PC2), respectively.^(^ ^3^, ^4^ ^)^ Genetic mutations can occur as both truncating and nontruncating in *PKD1* and *PKD2*, where mutation type has been linked to disease severity.^(^ ^2^ ^)^ Chronic kidney disease (CKD) and associated mineral bone disorders is associated with increased fracture rates^(^ ^5^, ^6^ ^)^ when compared with patients with ADPKD.^(^ ^7^ ^)^ In line with this, patients with ADPKD have suppressed bone turnover in advanced stages of CKD.^(^ ^7^, ^8^ ^)^ Intriguingly, even in the case of preserved kidney function, patients with ADPKD display low bone turnover with lower circulating bone alkaline phosphatase (ALP) levels, suggesting that bone phenotypes may be present prior to renal dysfunction.^(^ ^9^ ^)^ In contrast with other etiologies of CKD mineral bone disorders, low bone formation in ADPKD was not correlated with low bone volume and has lower fracture rates.^(^ ^8^, ^9^ ^)^ The molecular mechanisms, underlying how bone disease is propagated in patients who have CKD with *PKD* mutations versus patients who have non‐ADPKD CKD, remain to be completely understood.

The primary cilium plays a central role in bone biology where *PKD1/2‐*encoded polycystin (PC) proteins are expressed in both osteoblasts and osteocytes.^(^ ^10^, ^11^ ^)^ Bone formation and resorption is orchestrated by bone cell activities and responses to mechanical stress through complex cellular mechanisms.^(^ ^10^, ^12^, ^13^ ^)^ Cilia‐related signaling through the PC1/2 proteins has been linked to the regulation of fluid‐flow mechanosensation.^(^ ^14^, ^15^, ^16^, ^17^, ^18^ ^)^ Many cilia‐related signaling pathways are also implicated in ADPKD such as canonical Wnt signaling,^(^ ^19^, ^20^ ^)^ which plays an essential role in bone formation and is thought to activate PC1/2 heterodimerization in cilia.^(^ ^17^, ^20^, ^21^ ^)^ Animal models further underscore the importance of cilia in bone development as *PKD1* osteoblast‐specific conditional knockout models results in abnormal bone morphology, osteopenia,^(^ ^22^, ^23^, ^24^ ^)^ and low BMD.^(^ ^25^ ^)^ However, such *PKD* animal models differed from those observed in patients with ADPKD.^(^ ^26^ ^)^ Differences between both models are undefined, and whether the low bone turnover in ADPKD arises directly from altered cilia‐related signal transduction remains an open area of research.

ADPKD has been classified as a ciliopathy^,^ whereby the primary cilium operates as the major organelle associated with disease.^(^ ^27^, ^28^, ^29^ ^)^ The primary cilium is a transient structure that forms during a process called ciliogenesis. Dynamic regulation of cilia length is required for cilia‐related tissue activity and cellular signaling.^(^ ^30^ ^)^ Cilia elongation promotes mechanotransductive responses to strain through integrin signaling, whereas short cilia decrease intracellular cAMP and ion channel activity, which results in impaired fluid shear stress.^(^ ^31^ ^)^ Further, longer cilia were linked to tissue regeneration during injury whereby cilia‐emanating signaling was associated with enhanced proliferation.^(^ ^32^, ^33^ ^)^ Intriguingly, cystic ADPKD kidney cells have also been reported to increase rates of proliferation and growth.^(^ ^14^, ^34^ ^)^ Thus, it has even been proposed that cilia length may operate as an anticystogenic repair program that becomes aberrant in ADPKD and contributes cystic pathology.^(^ ^35^, ^36^, ^37^ ^)^ In this model, *PKD1/2* has been hypothesized to act as a brake on cilia signaling, and the loss or mutation of *PKD1/2* would lead to relentless signaling.^(^ ^15^ ^)^ Whether cilia defects similarly occur in human bone remains unknown.

Here, we utilized primary preosteoblast cells derived from patients with ADPKD and patients who have non‐ADPKD CKD to investigate for the first time primary cilia in human bone. Patients with non‐ADPKD CKD were chosen based on similar adynamic bone disease phenotypes as compared with ADPKD. Low bone turnover or adynamic bone is characterized by low bone formation rate per unit of bone surface (BFR/BS, μm^3^/μm^2^/y), reduced osteoblast and osteoclast cells, and the absence of osteoid tissue accumulation.^(^ ^5^ ^)^ Additionally, the selection criteria included CKD stage. Cilium elongation and induction were chosen as relevant endpoints for these initial investigations as they operate as surrogate markers for ciliogenesis and proper cilia regulation. Additionally, lithium chloride (LiCl), a known activator of cilia elongation, was used to assess cilia responsiveness. We report that primary cilia can be visualized in primary cultured human osteoblasts, and that control osteoblasts respond similarly to transformed cell lines. Osteoblasts derived from patients with *PKD* mutations displayed abnormal cilia that became significantly more elongated following LiCl treatment compared with healthy control cells. In contrast, cilia abnormalities of *PKD* mutant osteoblasts were distinct from those found in non‐ADPKD CKD osteoblasts, which further supports that distinct bone abnormalities are associated with ADPKD.

## Patients and Methods

### Patient data

Patients with ADPKD were selected from a cohort with glomerular filtration rates (GFRs) above 90 mL/min/1.73 m^2^. The bone formation rates (BFRs) of patients with ADPKD were consistent with low bone turnover or adynamic bone disease and were compared with patients who have non‐ADPKD CKD with similar bone turnover in early and advanced CKD stages as previously reported.^(^ ^7^, ^8^, ^9^ ^)^ Patient bone samples with mutations in either *PKD1* or *PKD2* (four patients) were chosen from both male and female subjects aged between 23 and 44 years. Importantly, *PKD* mutations included both truncating and nontruncating variations as mutation type has been linked to the rate of kidney disease progression.^(^ ^3^ ^)^ The BFRs/BSs (μm^3^/μm^2^/y) of the patients with ADPKD were *PKD1*‐trunkating (T; 0), *PKD1‐*nontrunkating (NT; 4.5), *PKD2‐T* (4.0), and *PKD2‐NT* (5.4). The patients with early‐stage non‐ADPKD CKD had BFRs/BSs (μm^3^/μm^2^/y) of 5.4 and 0.001, whereas those of healthy controls were 21.8 and 10.2. Replicate analyses were performed using cells derived from both male and female patients as healthy subjects. The total number of patients used for these analyses included two healthy controls, three patients with early‐stage non‐ADPKD CKD, two patients with late‐stage non‐ADPKD, and four patients with ADPKD with normal GFRs of 80 and 110 (mL/min/1.73 m^2^).

### Primary osteoblast cell isolation

Primary patient‐derived osteoblastic cells were cultured from bone biopsies as performed previously.^(^ ^38^ ^)^ Bone biopsies isolated from healthy volunteers were used as controls. Bone tissues were obtained from the anterior iliac crest using a bone biopsy trephine needle as previously described.^(^ ^38^ ^)^ Primary preosteoblasts were isolated from small fragments of cancellous bone and were cultured in DMEM supplemented with 20% fetal calf serum, 100‐mU/mL penicillin, 100‐mU/mL streptomycin, 250‐ng/mL amphotericin B (Invitrogen), and 100‐μg/mL ascorbic acid. Bone fragments were washed with fresh medium containing antibiotic–antimycotic solution, and fragments were minced into very small pieces, which were divided into six culture plates and cultured under standard 5% CO_2_ at 37°C. Upon cell attachment to plates and sufficient growth, the medium was changed twice weekly until 10–20 cell colonies were observed in each plate, which occurred within 1–4 weeks. Next, cell colonies were trypsinized, pooled, and subcultured. Upon reaching 80% confluence, the contents of the 19 plates were frozen in 20 mL of freezing medium composed of 10% DMSO and 30% FBS and stored in liquid nitrogen. Cells were harvested at equal confluences, and fed 24 hours prior to harvest to ensure reproducibility.

### Immunofluorescence, cell signaling, and cilia quantification

ADPKD, non‐ADPKD CKD, and healthy subject osteoblasts were plated at the same density and cultured until confluent. Osteoblast phenotypes were confirmed by staining with standard markers as previously performed.^(^ ^38^ ^)^ Cells were first incubated in serum‐free medium for 24 hours prior to treatments to induce growth arrest as previously performed.^(^ ^17^ ^)^ For cilia elongation experiments, cells were treated with either 10 or 25mM LiCl or no treatment (control) for 24 hours. Finally, cells were fixed in 4% paraformaldehyde and blocked with standard blocking buffer.^(^ ^38^ ^)^ Primary cilia were visualized using the antibodies acetylated tubulin and pericentrin; pericentrin marks the cilium base (Abcam). Cilia length was measured using pericentrin and acetylated tubulin. Cilia with lengths <1 μm were discarded. The number of ciliated cells were counted using CellSens software package (Olympus Corp); lengths measured per condition included 100–200 cells.

### Bone cell activity and mineralization assays

Biochemical parameters were determined using standard bioanalytical approaches. PKD mutation type in patients with ADPKD was determined either at the University of Colorado (Aurora, CO) or at the Mayo Clinic (Rochester, MN) by PCH.^(^ ^39^ ^)^ ALP activity in cells was measured by ELISA (Abbexa Ltd). The rate of mineralized matrix deposition of osteoblast cells was determined using differentiation cocktails and was assessed weekly by staining with 1% (w/v) Alizarin Red S (Sigma‐Aldrich).

## Results

### Primary cilia length and responsiveness is altered in ADPKD preosteoblastic cells

**Fig 1:**
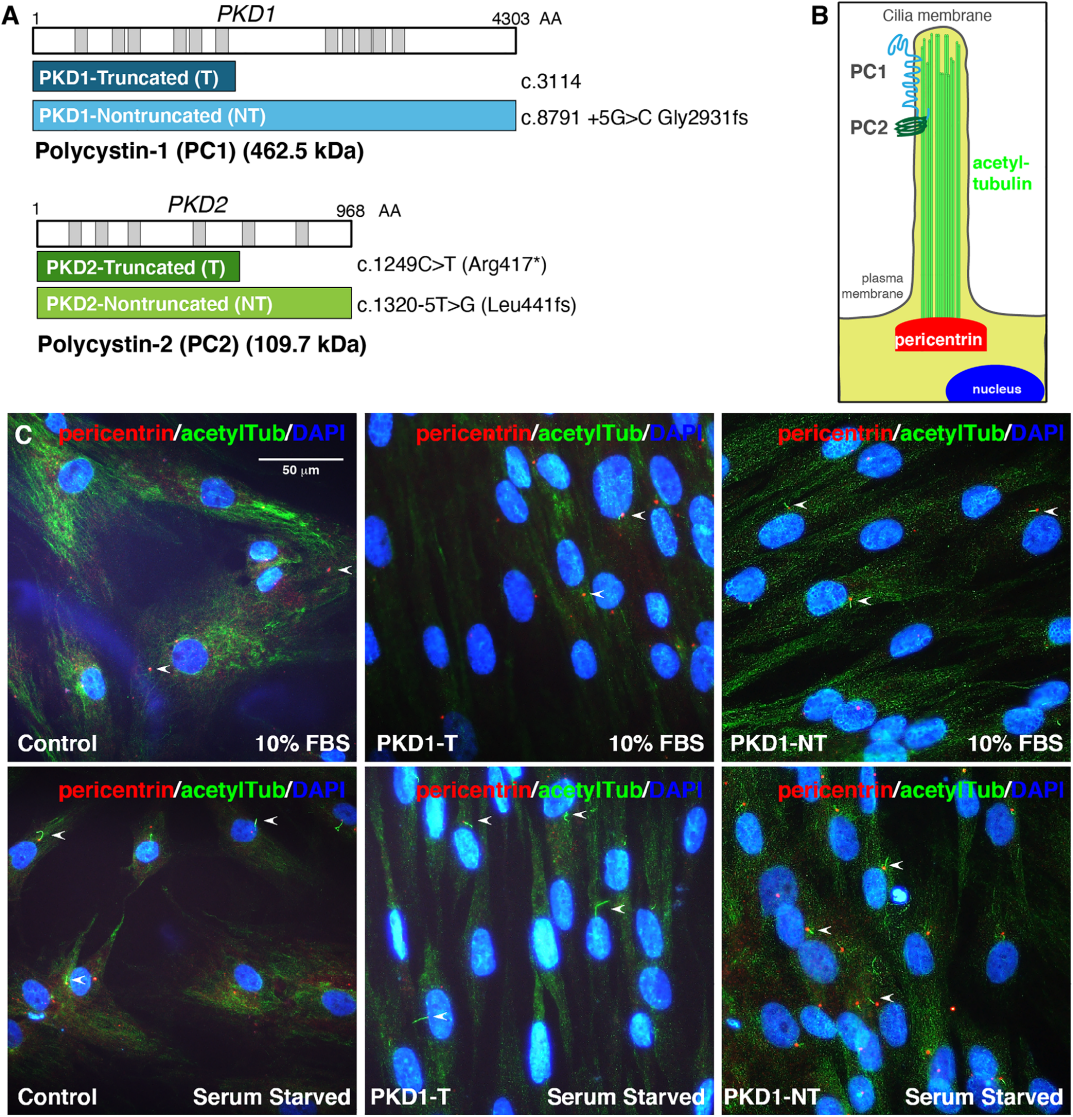
Primary cilia visualized in preosteoblasts derived from patients with autosomal dominant polycystic kidney disease (ADPKD). (*A*) Diagram of *PKD1* and *PKD2* genes encoding polycystin‐1 (PC‐1; 462.5 kDa) and polycystin‐2 (PC‐2; 109.7 kDa), respectively. The patients with ADPKD chosen were identified to contain truncating or nontruncating variations of both genes. *PKD1* mutations include truncation (navy) at codon 3114 or a nontruncated form (aqua) with mutations at codon 8791. *PKD2* mutations include truncation (dark green) at codon 1249 and nontruncated (light green) at codon 1320. (*B*) Cartoon depiction of primary cilia including PC‐1 and PC‐2 in the primary cilia, acetylated tubulin (green), and the basal body with pericentrin (red) at the base of the cilium. (*C*) Primary cilia (arrows) marked by pericentrin (red) and acetylated tubulin (green) in primary cultured osteoblasts from patients with ADPKD (*PKD1*) and healthy controls in 10% serum or serum‐starved conditions (24 hours), assessed by immunofluorescence staining; the nuclei were marked by DAPI (blue). Scale bar represents 50 μm. acetylTub = acetylated‐α‐tubulin; DAPI = 4,6‐diamidino‐2‐phenylindole.

Given the distinct differences in the bone of patients with ADPKD and *PKD* animal bone models, we examined whether preosteoblast cells derived from patients with ADPKD could serve as a model to study cilia in human bone. Preosteoblast cells were isolated from bone biopsies obtained from patients with ADPKD and patients with non‐ADPKD CKD with adynamic bone as defined histologically by low BFRs^(^ ^7^, ^8^, ^9^ ^)^ and biochemically by low‐circulating bone ALP levels.^(^ ^9^ ^)^ Patients with ADPKD showed confirmed mutations in either *PKD1* or *PKD2*.^(^ ^40^, ^41^, ^42^ ^)^ Disease progression of ADPKD is associated with the type of *PKD* mutation present. *PKD1* mutations have been associated with worse prognosis and earlier mortality than patients with *PKD2* mutations.^(^ ^40^, ^41^, ^42^ ^)^ Therefore, we selected both *PKD1* and *PKD2* with either truncating or nontruncating mutations (Fig. 1*A*). Patients with ADPKD were selected from a cohort with stage 1 CKD, defined by GFRs above 90 mL/min/1.73m^2^, to reduce potential off‐target effects of renal dysfunction for these initial bone analyses.

Primary cilia have been thoroughly examined in transformed cell lines and in animal models, but never in primary preosteoblasts isolated from human patients. To test whether cilia form properly, cultured osteoblasts were assessed in a series of immunofluorescence‐based assays. To first examine cilia induction, cells were cultured in complete medium and starved of serum for 24 hours as performed previously.^(^ ^43^ ^)^ To visualize cilia, cells were fixed and stained with antibodies targeting standard cilia markers of microtubules, with acetylated α‐tubulin and pericentrin (Fig. 1*B,C*).^(^ ^27^, ^44^ ^)^ Normal cell morphology was observed for osteoblasts of healthy cells and *PKD* mutant cells (Fig. 1*C*). Importantly, cilia induction by serum starvation was observed in all conditions (Fig. 1*C*,*D*), consistent with what has been well‐described in immortalized cell lines.^(^ ^14^ ^)^ Even in basal conditions prior to any treatment, *PKD* mutant cells showed longer cilia when compared with control cells.

**Fig 2:**
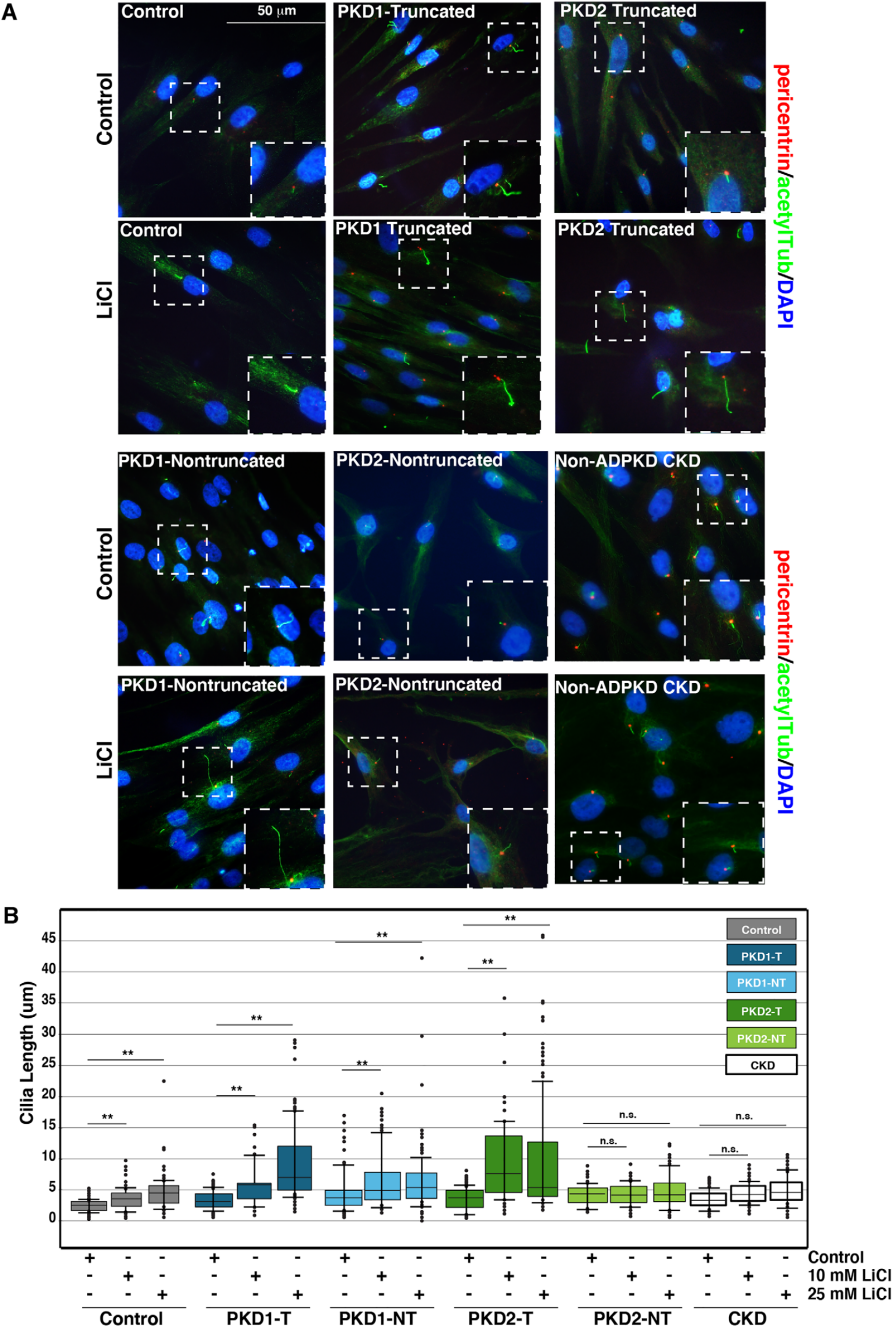
Primary cilia elongation by lithium chloride (LiCl) is enhanced in autosomal dominant polycystic kidney disease (ADPKD) osteoblasts. (*A*) Primary cultured osteoblasts from patients with ADPKD, non‐ADPKD chronic kidney disease (CKD), and healthy controls treated with 10mM LiCl or control medium and stained with the antibodies pericentrin (red) and acetylated tubulin (green) for immunofluorescence analyses. Nuclei were marked by 4,6‐diamidino‐2‐phenylindole (DAPI; blue). White‐dashed boxes mark representative cilia from each condition: healthy controls, *PKD1*‐truncated (*PKD1‐T*), *PKD1*‐nontruncated (*PKD1‐NT*), *PKD2*‐truncated (*PKD2‐T*), and *PKD2*‐nontruncated (*PKD2‐NT*). (*B*) Scatter‐plot quantification of cilia length following 10mM LiCl, 25mM LiCl, and control treatments in serum‐starved osteoblasts from patients with ADPKD and non‐ADPKD CKD, and healthy controls. Healthy cilia increased from 2.6 ± 0.1 μm in serum‐starved cells to 3.5 ± 0.2 μm with 10mM LiCl and 4.95 ± 0.3 μm with 25mM LiCl (**p < 0.001). *PKD1‐T* cilia increased from 3.9 ± 0.2 μm in control treatments to 5.4 ± 0.3 μm with 10mM LiCl and 9.4 ± 0.5 μm with 25mM LiCl (**p < 0.001). *PKD1‐NT* cilia increased from 4.2 ± 0.2 μm to 5.5 ± 0.3 μm and 8.7 ± 0.7 μm (**p < 0.001). *PKD2‐T* cilia increased from 3.7 ± 0.2 μm in control treatments to 7.3 ± 0.4 μm with 10mM LiCl and 7.4 ± 0.4 μm with 25mM LiCl (**p < 0.001). *PKD2‐NT* cilia did not significantly increase following LiCl treatment (4.4 ± 0.2 μm in controls; 4.3 ± 0.2 μm with 10mM LiCl; 5.1 ± 0.2 μm with 25mM LiCl; **p < 0.001). Error bars indicate mean ± SEM (*n* > 200). *p* Values were calculated by Bonferroni corrected pairwise comparisons. Scale bar = 50 μm.

As dynamic regulation of cilia length is essential for cellular and mechanotransductive signaling,^(^ ^30^ ^)^ we next assessed cilia elongation as an initial indicator of cilia responsiveness. Cells were treated with LiCl as it induces cilia elongation in mice osteoblasts.^(^ ^45^ ^)^ Notably, LiCl activates canonical Wnt signaling, a pathway previously implicated in cyst expansion of polycystic disease in animal models, through the inhibition of glycogen synthase kinase 3.^(^ ^45^ ^)^ Following serum starvation, cells were treated with either 10mM or 25mM LiCl for 24 hours and processed for immunofluorescence analyses. Cilia length was measured using pericentrin to mark the cilium base acetylated α‐tubulin as microtubules extend to the tip of the organelle. Ciliated cell numbers were unchanged in all conditions. In control cells, cilia elongation was initiated as expected, increasing in a dose‐responsive manner by 0.9 μm and 2.4 μm in 10mM and 25mM LiCl treatments, respectively (Fig. 2*A*,*B*). Similar baseline cilia lengths were observed across ADPKD‐derived osteoblasts; however, levels of cilia responsiveness were highly varied across different *PKD* mutations. Nontruncating *PKD2* mutated osteoblasts were not significantly increased in response to LiCl treatments, increasing by only 0.7 μm with 25mM LiCl (not significant; Fig. 2*B*). Strikingly, in osteoblasts with truncating *PKD2* and *PKD1* mutations, cilia showed significantly higher responses to LiCl, where cilia length increased by 3.75 μm and 5.49 μm, respectively (Fig. 2*A,B*). Osteoblasts with nontruncating *PKD1* mutations also increased significantly more than controls: by 4.5 μm with 25mM LiCl (Fig. 2*A,B*). It is interesting to note that the highest levels of abnormal cilia elongation occur with truncating mutations in *PKD1* as these have been correlated with the worse prognosis in patients.

Given the similarities of adynamic bone defects and histology, we next assessed cilia responsiveness in non‐ADPKD CKD patient osteoblasts. In contrast, cilia elongation was not significantly increased in non‐ADPKD CKD cells where cilia length was only increased by 1.1 μm following treatment with 25mM LiCl (Fig. 2*B*). That cilia elongation was significantly higher in ADPKD relative to osteoblasts from other types of CKDs suggests that ciliary dysregulation is related to *PKD* mutations and supports a model whereby cilia defects may be linked to molecular mechanisms underlying the distinct low bone turnover in ADPKD.

### ADPKD osteoblasts accelerate mineralized matrix deposition when compared with osteoblasts from patients with non‐ADPKD CKD and adynamic bone disease

**Fig 3:**
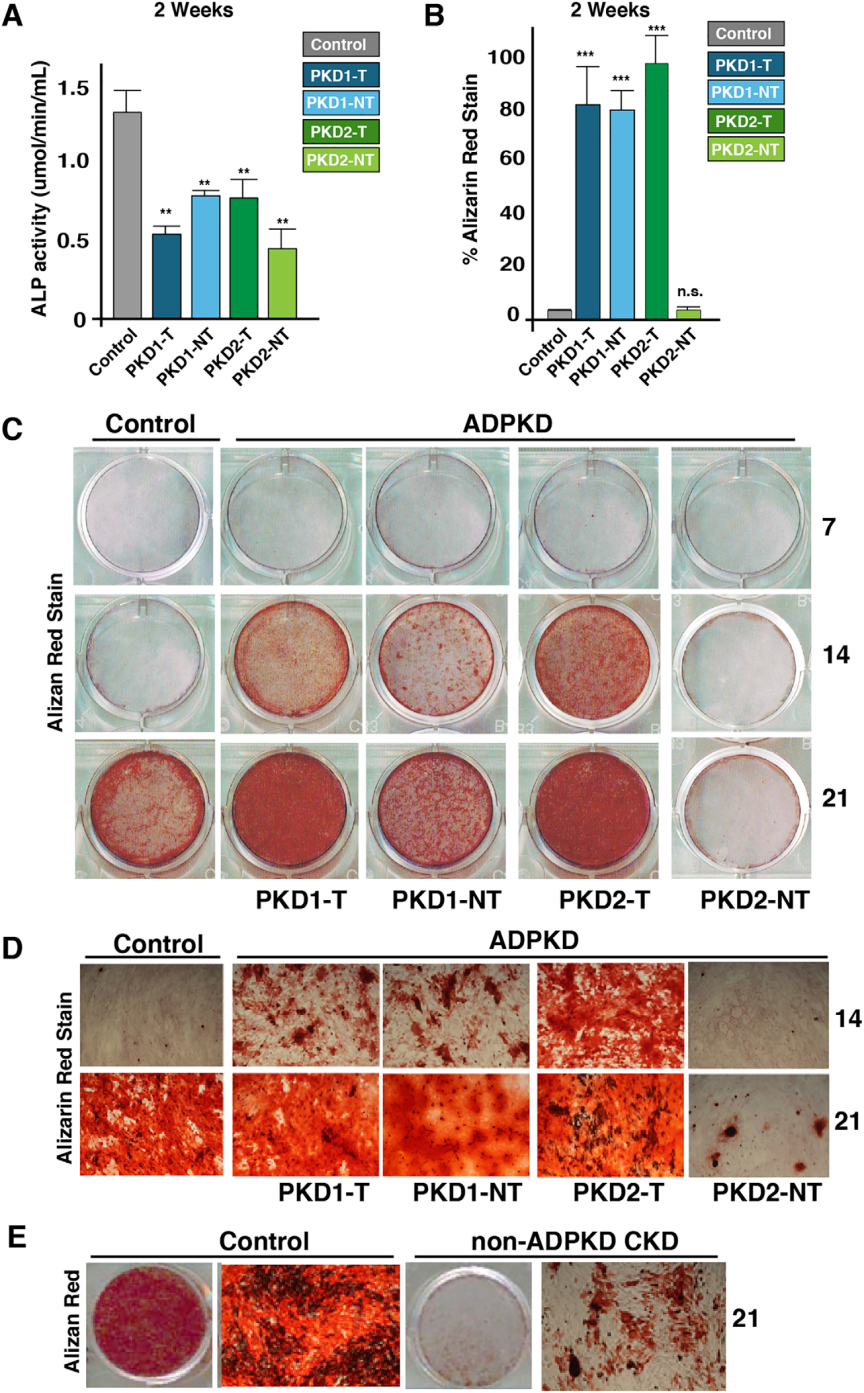
Autosomal dominant polycystic kidney disease (ADPKD) osteoblasts bone mineralize more rapidly in culture compared with healthy controls. (*A*) Cultured *PKD* mutant osteoblasts exhibit low alkaline phosphatase (ALP) activities (μmol/min/mL) after 14 days in culture. (*B*,*C*) Time‐course analyses of ADPKD osteoblast mineralization compared with healthy controls after 7, 14, and 21 days assessed by Alizarin Red S staining. Cells were grown under mineralizing conditions (10mM‐β‐glycerol phosphate and 100‐μg/mL ascorbic acid). Osteoblasts with nontruncating mutations in *PKD1/2* showed lowest levels of mineralization, whereas truncating *PKD1/2* mutations mineralized at levels similar to or higher than healthy controls. Quantification of mineralization rates were assessed by Alizarin Red S staining after 2 weeks. (*D*) Alizarin Red S stained mineralized nodules under light microcopy (×40) at 14 and 21 days from mutant *PKD* osteoblasts. (*E*) Osteoblasts from patients with non‐ADPKD chronic kidney disease (CKD) with low bone turnover mineralize slower than healthy osteoblasts after 21 days as assessed by Alizarin Red S staining intensity. Images displayed are representative of replicate experiments (*n* > 3). *PKD1‐T* = *PKD1‐*truncated; *PKD1‐NT = PKD1‐*nontruncated; *PKD2‐T = PKD2‐*truncated; *PKD2‐NT = PKD2‐*nontruncated. Error bars indicate mean ± SEM. **p > 0.01 and ***p > 0.001.

Low bone turnover in CKD is associated with higher fracture rates and decreased mineralization.^(^ ^38^ ^)^ Circulating levels of bone ALP is an indicator of osteoblastic activity and has been reported to be low in patients with ADPKD.^(^ ^8^, ^9^ ^)^ Consistent with this, healthy controls had circulating bone ALP levels of 20 (μg/L) and total circulating ALP levels of 70 (IU/L). In contrast, ADPKD bone ALP levels were lower in all four patients with *PKD1‐T* (9.6 μg/L), *PKD1‐NT* (6.8 μg/L), *PKD2‐T* (8.4 μg/L), and *PKD2‐NT* (9.6 μg/L). Total ALP levels across this ADPKD cohort were *PKD1‐T* (42 IU/L), *PKD1‐NT* (35 IU/L), *PKD2‐T* (44 IU/L), and *PKD2‐NT* (40 IU/L). Next, we assessed osteoblast activity by monitoring ALP in cultured cells from our ADPKD cohort with low bone ALP. Compared with healthy control cells, ADPKD osteoblasts showed lowered ALP activity as in previous reports of low ALP levels in cultured osteoblasts from non‐ADPKD CKD (Fig. 3*A*).^(^ ^46^ ^)^ Next, we examined Alizarin Red in cultured ADPKD osteoblasts as a marker of mineralized matrix deposition. Cells were plated at equal density and maintained in an osteoblast differentiation cocktail of ascorbic acid, β‐glycerol phosphate, and dexamethasone as previously performed.^(^ ^38^ ^)^ Time‐course analyses, which assessed Alizarin Red S staining over the course of 21 days, revealed that healthy control cells began to show visible mineralization after 14 days and continued to increase through 21 days (Fig. 3*B*–*D*). Osteoblasts with truncating *PKD1/2* mutations showed significantly higher levels of Alizarin Red S staining at both the 14‐ and 21‐day time points, exceeding levels of control cells (Fig. 3*B*–*D*). Increased mineralized nodules were also apparent in *PKD1/2* truncated osteoblasts, visualized under light microscopy (Fig. 3*C*). In contrast, osteoblasts with *PKD2* nontruncating mutations showed lower staining by Alizarin Red S throughout all time points. Similarly, osteoblasts derived from patients with non‐ADPKD CKD also showed lower levels of Alizarin Red S staining compared with healthy controls, consistent with previous reports (Fig. 3*E*). Thus, these data suggest that the *PKD* mutation type may be correlated with specific rates of mineralized matrix deposition, as was the case with cilia responsiveness. Furthermore, though both patients with ADPKD and non‐ADPKD CKD have low bone turnover, important differences in bone quality such as fractures exist in both groups.^(^ ^9^ ^)^ Whether primary cilia are responsible for the differences in bone from patients with CKD with or without *PKD* mutations remains an open area of research that warrants larger‐scale studies with more patients.

## Discussion

**Fig 4:**
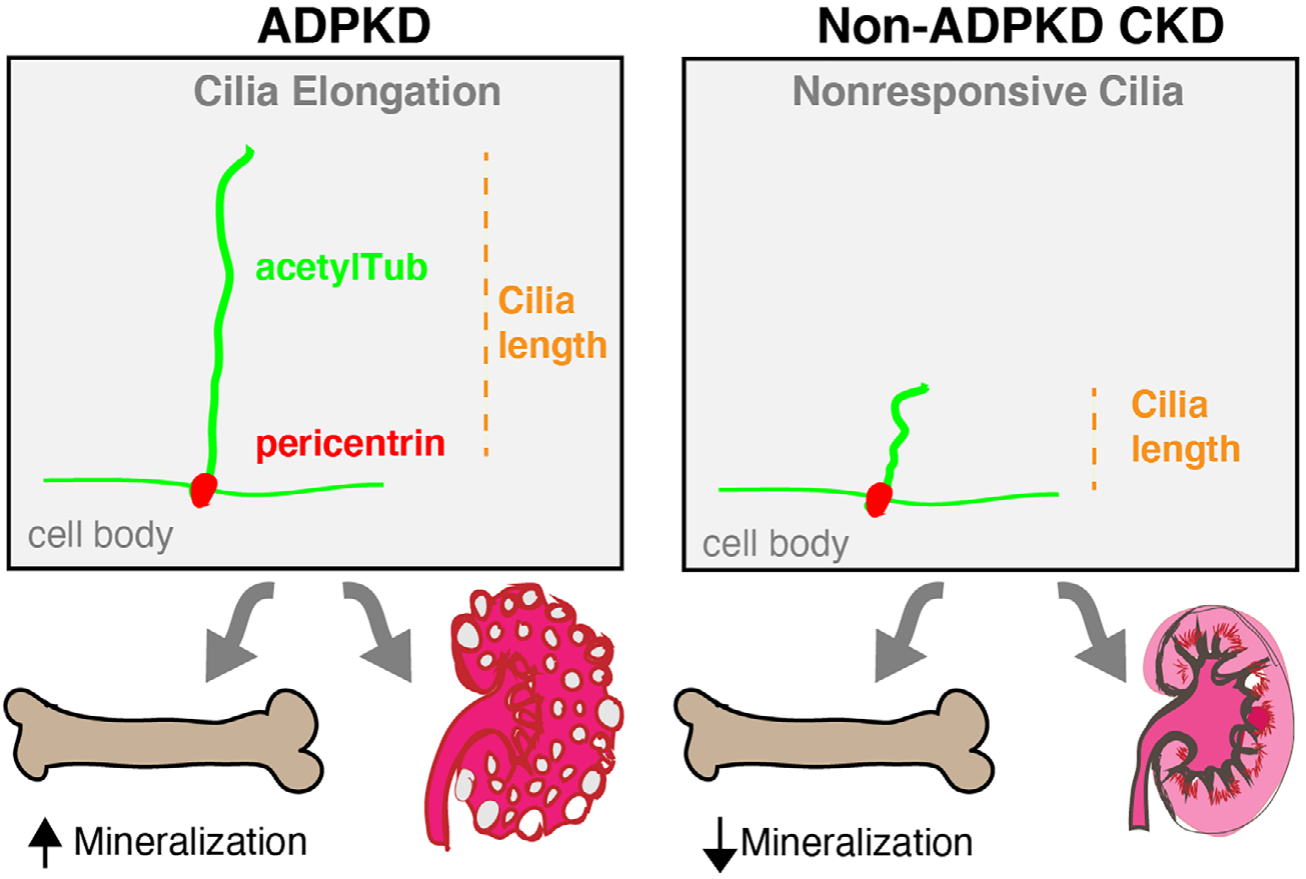
. Adynamic bone in autosomal dominant polycystic kidney disease (ADPKD) exhibits distinct features when compared with adynamic bone from patients with non‐ADPKD chronic kidney disease (CKD). Primary osteoblasts derived from patients with ADPKD showed altered cilia responsiveness, marked by elongation to lithium chloride treatment, and corresponded to differences in bone cell activities including the rates of bone mineral deposition. acetylTub = acetylated‐α‐tubulin.

In sum, we report that osteoblasts derived from patients with ADPKD represent a key resource for studies of cilia and *PKD* mutations, where bone phenotypes are not well‐represented in animal models.^(^ ^8^, ^9^ ^)^ Low‐circulating ALP levels in patients with ADPKD were recapitulated in cultured primary osteoblasts, and cilia induction was similar to that seen with immortalized cell lines, supporting the utility of this system for future large‐scale analyses. Specifically, patient‐derived osteoblasts could provide an important way into the investigation of responses of human cells to new cilia‐targeted treatments that reduced cyst formation in mice.^(^ ^47^, ^48^ ^)^ Using this approach, we uncovered that different *PKD* mutation types resulted in distinct cilia responsiveness and accelerated mineralized matrix deposition when compared with osteoblasts derived from patients with non‐ADPKD CKD with adynamic bone (Fig. 4). Further, the defective cilia signaling in ADPKD cells showed increased Alizarin Red staining, and mineralized nodules suggest that *PKD1* mutations and *PKD2‐T* mutations correlate with increased mineralized matrix deposition. However, future studies on the relative contributions of preosteoblast differentiation into mature osteoblasts in relation to mineralization could uncover new insights.

Genotype–phenotype studies of ADPKD have the potential to advance diagnostics and therapeutic strategies. For example, mutations in *PKD1* are more prevalent and lead to more severe kidney disease when compared with *PKD2* mutations. Furthermore, truncations of *PKD1* are associated with greater deterioration of kidney function than nontruncating variations of *PKD1*. Our data support a model whereby truncating or nontruncating mutations in *PKD1* and *PKD2* lead to differential responsiveness of cilia in osteoblasts. We report that *PKD2* nontruncating mutations showed the least responsive cilia in the presence of LiCl when compared with truncating *PKD2* or *PKD1* mutations. Wnt signaling activity is linked to kidney disease in ADPKD.^(^ ^19^, ^20^ ^)^ As LiCl is a known activator of Wnt signaling, our data support a model whereby *PKD1*‐truncating mutations, which are associated with more severe kidney disease, lead to osteoblasts with the most‐responsive cilia and accelerated mineralized matrix deposition rates. In contrast, the *PKD2* nontruncating mutations that are associated with the mildest kidney disease would not exhibit the exaggerated sensitivity to Wnt inhibition. Future work comparing the molecular signaling cascades across tissues with truncating *PKD* mutations, such as frameshifting indels, nonsense, and splicing; or nontruncating *PKD* mutations from missense or in‐frame indels could lead to new insights into genetic‐based prognosis and targeted therapy.

Studies including an increased number of patients will be required to definitively assess whether *PKD* mutation types globally determine cilia functions in bone. Because bone defects in ADPKD are already present with normal renal function,^(^ ^9^ ^)^ future work on the molecular players linking cilia and bone health may help identify future therapies. The sensitivity of PKD‐mutated osteoblasts to LiCl implicates the Wnt signaling pathway, which is consistent with reports of Wnt signaling activity in ADPKD kidneys. Finally, we propose that the use of primary non‐ADPKD CKD osteoblasts also provides an approach for understanding the bone disease across CKD etiologies, which could have important implications for the underlying mechanisms of crosstalk between the kidney and bone.

## AUTHOR CONTRIBUTIONS

**Renata Pereira:** Conceptualization; data curation; formal analysis; investigation; methodology; writing‐review & editing. **Berenice Gitomer:** Resources. **Michel Choncol:** Resources. **Peter Harris:** Resources. **Kathleen Noche:** Data curation. **Isidro Salusky:** Conceptualization; investigation; project administration; resources; supervision; writing‐review & editing. **Lauren Albrecht:** Formal analysis; writing‐original draft; writing‐review & editing.

### PEER REVIEW

The peer review history for this article is available at https://publons.com/publon/10.1002/jbm4.10464.
